# Supplementary material for: A Tumor Homing Peptide-Linked Arsenic Compound Inhibits Pancreatic Cancer Growth and Enhances the Inhibitory Effect of Gemcitabine
Source: Int J Mol Sci. 2024 Oct 22;25(21):11366. doi: 10.3390/ijms252111366 (PMC11546692; doi:10.3390/ijms252111366)
Supplement: Supplementary file 1 [file ijms-25-11366-s001.zip › ijms-3232115-supplementary.pdf]

Supplementary Materials:

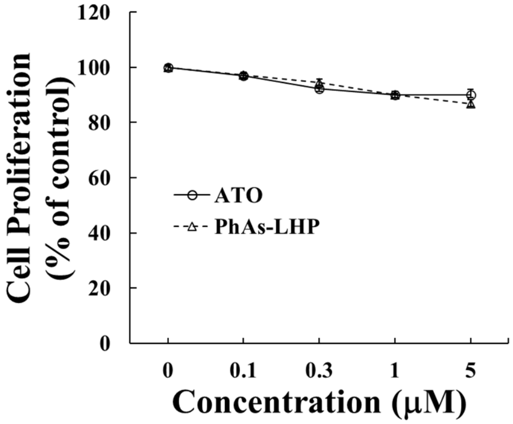

Supplementary Fig.S1. The effects of arsenic acid or the peptide-linked arsenic compound on the proliferation of normal pancreatic epithelial cells. Normal pancreatic epithelial cell (HPDE) was incubated with arsenic acid (ATO) and the peptide-linked arsenic compound (PhAs-LHP) for 24 h. The cell proliferation was determined by MTT. ATO and PhAs-LHP significantly inhibited the proliferation of HPDE by 10% at 5 ◉M. There was no significant difference between the effect of ATO and PhAs-LHP.

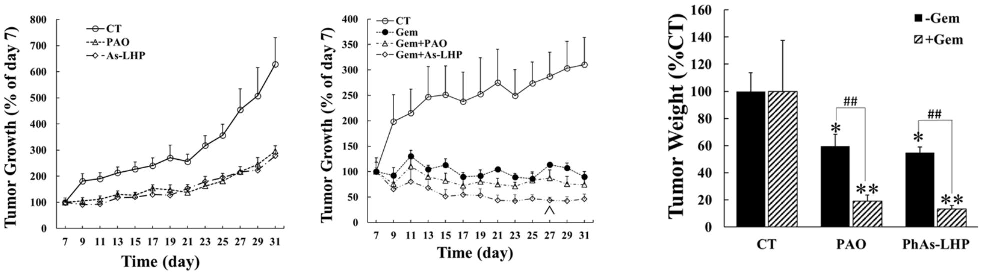

Supplementary Fig.S2. The inhibition by PAO and PhAs-LHP of pancreatic cancer growth was increased by gemcitabine. Tumor growth was calculated by taking the tumor volume measured on Day 7 when the treatment was started as 100% (A, B). Arsenic acid (PAO) and the peptide-linked arsenic compound (PhAs-LHP) decreased the tumor growth (A). Gemcitabine decreased the tumor growth and appeared to further reduce the tumor growth of mice treated by PAO and PhAs-LHP (B). Relative tumor weights were calculated taking control (not treated by either PAO or PhAs-LHP) as 100% (C). PAO and PhAs-LHP decreased the tumor weight significantly compared to the nontreated control (CT). In the presence of gemcitabine, the tumor weights from the mice treated with PAO or PhAs-LHP were further reduced compared to gemcitabine alone (C). ^, *, $p<0.05$; **, ##, $p<0.01$.

Supplementary method

Determination of arsenic distribution and concentration by GFAAS

1. Materials. The commercially available reagents used in this work were of analytical grade or higher purity, as specified. Milli-Q water (18.2 MΩ/cm resistivity, Millipore, Merk Millipore, Burlington, USA) was used for all solutions as indicated. Pyroneg detergent was sourced from Diversey Lever Australia Pty Ltd (Smithfield, Australia). Ultrapur nitric acid (60% v/v, <1.0 ppb

As batch analysis) and tetramethylammonium hydroxide (TMAH, 25% v/v) was purchased from Merck (Darmstadt, Germany). Analytical volumetric flasks (Class A, accuracy: ± 0.04 mL) were procured from Glassco (Dandenong South, Australia). A certified arsenic standard solution (1000 mg/mL, 10% HNO_3, Lot #R2-As688291), and the palladium ($Pd(NO_3)_2$, 5 mg/mL) and magnesium ($Mg(NO_3)_2$, 10 mg/mL) matrix modifier solutions were obtained from Astral Scientific (Taren Point, Australia). An arsenic electrodeless discharge lamp and standard transversely heated graphite atomiser tubes were obtained from Perkin Elmer Instruments (Melbourne, Australia).

2. Tissue sample preparation. Prior to analysis the mouse tissue was freeze-dried (Alpha 3-4, Christ, Germany) overnight. The freeze-dried tissue samples (approximately 25 mg for kidney and liver samples, and 5 mg for brain and heart samples) were then digested in TMAH (25 % v/v, 1.00 mL (kidney and liver samples), or 200 µL (brain and heart samples)) at room temperature (approx. 20-22 °C), until complete digestion was achieved (1-2 days). The resulting solutions were transferred to pre-soaked (Pyroneg, 3 days; 1.5 M HNO_3. 3 days; Milli-Q water, 3 days; rinsed Milli-Q water, 5×) volumetric flasks and diluted to 25.00 mL (kidney and liver samples) or 5.00 mL (brain and heart samples). Triplicate samples were made for each organ tissue sample. Extra samples from the control group tissues were made for instrument optimisation.

3. Analysis of As in tissue samples by GFAAS. The As present in each sample was quantified using a Perkin Elmer PinAAcle 900Z THGA longitudinal Zeeman graphite furnace instrument equipped with an AS-900 autosampler and an AA Accessory cooling system. The instrument was operated using Syngistix for AA Software. An arsenic electrodeless discharge lamp set at a wavelength of 193.7 nm and a slit width of 0.7 nm, typically produced an instrument energy reading of approximately 63 W. Arsenic was atomised from the surface of the pyrolytic graphite-coated tube using the furnace conditions listed in Table 1. Each analysis was performed on a total volume of 25 µL, containing the chemical matrix modifier (5 µL, 1 mg/mL Pd, 0.6 mg/mL $Mg(NO_3)_2$), and the matrix matched spike or sample solution (20 µL). The analyte reading was obtained during the atomisation step (2000 °C). Argon gas was supplied for the duration of the analysis.

Table S1: Graphite furnace operating conditions.

Stage	Temperature (°C)	Ramp (s)	Hold (s)	Gas flow (mL.min-1)
Dry 1	110	1	30	250
Dry 2	130	15	30	250
Ash	1200	10	20	250
Atomisation	2000	0	5	0
Clean Out	2450	1	3	250

Arsenic standard solutions were prepared using a certified As standard solution in tissue sample digests (from control sample organs, in order to match the matrix of the samples to be analysed) to produce calibration curves (0-80.00 µg As/L, R^2 = 0.997 – 1.00). Spiked samples (30 and 50 µg As/L) were prepared in the same manner as the standard solutions and were used intermittently throughout the analysis as a quality control.
